# Transforming Care Through Co-Design: Developing Inclusive Caregiver-Centered Education in Healthcare

**DOI:** 10.3390/healthcare13030254

**Published:** 2025-01-27

**Authors:** Jasneet Parmar, Tanya L’Heureux, Richard Lewanczuk, Jonathan Lee, Lesley Charles, Laurel Sproule, Isabel Henderson, Esha Ray Chaudhuri, Jim Berry, Kimberly Shapkin, Linda Powell, David Nicholas, Glenda Tarnowski, Myles Leslie, Michelle Lobchuk, Joanne Kaattari, Ambere Porter, Vivian Ewa, Linda Podlosky, Jacqueline Pei, Sarah Mosaico, Jamie Penner, Shannon Saunders, Sharon Anderson

**Affiliations:** 1 Department of Family Medicine, University of Alberta, Edmonton, AB T6G 2T4, Canada; jasneet.parmar@ualberta.ca (J.P.); trpankiw@ualberta.ca (T.L.);; 2 Alberta Health Services, Edmonton, AB T5J 3E4, Canada; rlewancz@ualberta.ca (R.L.);; 3 Ornge Air Ambulance, Mississauga, ON L4W 5H8, Canada; 4 Family Caregiver, Edmonton, AB T6G 2T4, Canada; 5 Faculty of Nursing, University of Calgary, Calgary, AB T2N 4L6, Canada; kimberly.shapkin@ucalgary.ca; 6 Faculty of Social Work, University of Calgary, Calgary, AB T2N 1N4, Canada; nicholas@ucalgary.ca; 7 Department Community Health Sciences, Cumming School of Medicine, University of Calgary, Calgary, AB T2N 4Z6, Canada; myles.leslie@ucalgary.ca; 8 Rady Faculty of Health Sciences, College of Nursing, University of Manitoba, Winnipeg, MB R3T 2N2, Canada; michelle.lobchuk@umanitoba.ca (M.L.); jamie.penner@umanitoba.ca (J.P.); 9 Alzheimer Society of Alberta and Northwest Territories, Edmonton, AB T6H 1M4, Canada; 10 Department of Family Medicine, University of Calgary, Calgary, AB T1Y 5S3, Canada; vivian.ewa@albertahealthservices.ca; 11 Department of Pediatrics, College of Social Sciences and Humanities, University of Alberta, Edmonton, AB T6G 1C9, Canada; jpei@ualberta.ca; 12 SPEECHified, Edmonton, AB T8A 4T7, Canada; sarahmosaico@speechified.org

**Keywords:** co-design, family caregivers, caregiver-centered care, health education, inclusive collaboration, systemic change

## Abstract

**Background**: Family caregivers provide most (75–90%) of the essential unpaid care and support for individuals living with chronic conditions, disabilities, and age-related needs in the community, with about half performing medical tasks traditionally performed by professionals. Caregivers also assist with 15 to 35% of the care in congregate care settings. Yet despite their critical contributions to patient care, caregivers face stress, declining well-being, and insufficient recognition in healthcare systems. Addressing these challenges requires innovative, person-centered approaches to training healthcare providers. Co-design or co-production are participatory research methods that involve individuals with lived experience to ensure relevance and impact. **Objective**: This study sought to understand how participatory co-design principles influenced learning, collaboration, and engagement among diverse participants in developing a caregiver-centered education program for healthcare providers. Actionable recommendations for optimizing co-design processes are provided. **Methods**: Eighty-five participants from a team of 155 collaborators, including caregivers, healthcare providers, educators, policymakers, and leaders, participated in ten focus group sessions conducted in Zoom breakout rooms. Interviews were transcribed verbatim and analyzed using Thorne’s interpretive description and Braun and Clarke’s reflexive thematic analysis. **Results**: Participants described the co-design process as fostering collaboration, inclusivity, and skill enhancement. Exposure to diverse perspectives expanded transformative understanding and prompted reflection on caregiver support within professional practices. Skilled facilitation ensured equitable engagement. Challenges included information overload and personal time constraints. Participants liked using breakout rooms to mitigate the dynamics of large group management. Still, they recommended pre-meeting materials, flexible scheduling, and expanding stakeholder diversity (e.g., rural, Indigenous, and immigrant caregivers). **Conclusions**: Co-design fosters meaningful, caregiver-centered education through collaboration and inclusivity. Addressing logistical challenges and representation gaps can further enhance the impact of co-design and empower multi-level, interdisciplinary partners to inform equitable healthcare education.

## 1. Introduction

Family caregivers form the foundation of the healthcare system, delivering 75–90% of essential care and support for individuals with chronic conditions, disabilities, or age-related needs living in community settings [1,2]. Additionally, they contribute to 15–35% of the care provided in congregate care environments [3]. The Columbia Mailman School of Public Health recently reported that in the United States, 44.58 million family caregivers provide unpaid labor valued at approximately USD 873.5 billion annually, representing 3.2% of the GDP. This valuation, derived using the Proxy Goods method, substitutes caregiver hours with equivalent formal worker hours, highlighting the immense economic contribution of caregivers and the irreplaceable nature of their support [4]. They perform unpaid work that encompasses emotional, physical, and practical care, often in complex and evolving family dynamics shaped by care, social support, diversity, economic conditions, and geographic location [5,6]. 

In this paper we will use caregiver, who might also be referred to as a family caregiver, carer, care partner, or designated support person, defined as anyone who takes on unpaid work providing emotional, physical, or practical support to a family member, chosen family, friend, or neighbor facing challenges related to mental or physical illness, disability, or age-related needs [7].

The challenges caregivers face can be significant. Due to medical advances, shorter hospital stays, and longer life expectancies, caregiving has grown more complex over the years. About half of caregivers are now doing medical care tasks that can only be performed by regulated health professionals in medical settings [8]. These changes, combined with modern family dynamics—such as smaller households, family members living further apart, and more dual-income families—have placed greater demands on caregivers [2,9,10]. 

Research reports increasing stress, anxiety, depression, burnout, and declining well-being amongst caregivers [11,12]. However, it is not giving care per se that is stressful. It is being overloaded with work and worry. Distress rises sharply for caregivers who are caring more than 20 h per week and/or for someone with dementia, depression, anger, responsive behaviors, and severe impairments [13]. Caregiving is deeply meaningful for many, offering purpose and connection. Indigenous caregivers, for example, describe caregiving as completing the circle of life, a cultural act that passes wisdom and relationships to the next generation [14,15,16].

Despite their essential contributions, caregivers are often overlooked within the healthcare system, receiving little recognition or support [17,18]. Frequently, they are excluded from care planning, communication, and decision-making processes. Less than a quarter are asked about their caregiving, and even fewer about their health or well-being [19,20]. This lack of acknowledgment can exacerbate caregivers’ feelings of isolation and undervalue their critical role in ensuring patient well-being and system sustainability [20,21]. The lack of recognition, communication, and partnering from health providers leaves a critical gap in caregiver support [17,18,22,23]. This gap has led to calls for more person-centered approaches that view caregivers as integral partners in care [24,25]. The Institute for Healthcare Improvement Quintuple Aim [26], the WHO framework on people-centered and integrated health services [27], and major family caregiver advocacy groups like the AARP [28] and the Canadian Centre for Caregiving Excellence [29] emphasize the importance of partnerships and co-design with caregivers. These frameworks call for a shift from health systems focused on diseases and institutions to ones centered on people and communities, ensuring equitable, high-quality, and responsive care for all.

Healthcare workers need more knowledge, skills, and approaches to increase their comfort and confidence to engage caregivers effectively, leaving a critical gap in caregiver support [17,18,22,23]. Addressing this challenge requires innovative approaches to training healthcare providers to engage with and support caregivers, reimagining them as partners as systems are transformed to be more inclusive and responsive to caregivers [17,18,22,23].

### 1.1. The Role of Co-Design and Co-Production

Co-design and co-production are participatory research, practice, and quality improvement approaches that empower individuals with lived experiences to shape education, services, and policies [30,31,32]. Co-production spans the entire lifecycle—from planning to evaluation—while co-design focuses on developing solutions tailored to participant needs [32]. Both approaches integrate lived experiences into decision-making, fostering systemic change by challenging assumptions, addressing stigma, and building trust [33,34].

Co-design and co-production can drive systemic change by challenging assumptions, raising awareness, and shifting mindsets [31]. As practices, they empower participants by giving them a direct voice in shaping education, services, and policies. They also ensure that lived experiences are integrated into solutions, fostering, in the present case, both a deeper understanding of the complexities of caregiving amongst healthcare workers and a greater sense of engagement and participation amongst family caregivers. In this way the processes enhance the relevance of solutions and build trust and shared ownership, helping dismantle barriers like stigma, cultural biases, and inequities in care [31,34,35,36].

### 1.2. Developing the Caregiver-Centered Care Education (CCCE)

The origins of this education initiative date back to symposia held between 2014 and 2017, where caregivers, health and community care providers, educators, and researchers identified a critical need for targeted education for healthcare workers to support caregivers [37,38,39,40,41,42]. In the words of one family caregiver, “[the system] always focus[es] on education for family caregivers. Stop trying to build a better caregiver and educate providers to support us”. Aligned with this front-line perspective, researchers were also starting to recommend competency-based education for healthcare workers [22,43]. Symposium participants highlighted critical gaps where caregivers were often unrecognized and excluded from care processes. They called for training to help healthcare workers better recognize caregivers’ roles, communicate effectively, foster resilience, assist in navigating systems, and improve the culture of caregiving support [37,38,39].

These recommendations led to the creation of the caregiver-centered care competency framework that used co-design principles to select out the knowledge, skills, and attitudes needed to effectively support caregivers [40,44]. Six competency domains were identified, and the competency framework would become the North Star for the next co-design phase in which the CCCE intervention was created [44].

The CCCE consists of three levels [44]. The foundational level is a one-hour module that introduces all six competency domains. It is co-designed for all health and community care workers, regulated and non-regulated. Learners who have completed this training since its launch in 2020 report increased knowledge and confidence in working with caregivers [45]. The Advanced level offers six one-hour modules, each exploring one of the competency domains in depth [46]. It is designed for health and community care providers whose scopes of practice or positions require considerable interaction with caregivers. The champions level, three one-hour modules, equips participants to lead caregiver-centered care initiatives through three modules focused on inspiring, leading, and managing change. Each level integrates person-centered principles, drawing on lived experiences of caregivers and providers, best practices in interdisciplinary education [47,48,49,50,51], and interactive learning methods [52,53]. The co-design of the education is described in those publications.

Although the work is reported here as a single study, it is, in fact, a longitudinal co-design process spanning nearly a decade. We have continued to refine and evaluate each caregiver-centered care component, that is person-centered care for caregivers. Some of the original participants from the 2014 consultations (n = 16) remain engaged, reflecting the initiative’s sustained collaborative nature. In this paper, we focus specifically on current participants’ perceptions of the co-design process in team meetings.

### 1.3. Exploring the Impact of Co-Design

We sought to understand the following: (1) the participant’s experiences of being part of the co-design team and participants’ recommendations for improvements. While co-design has become an increasingly popular approach, understanding its impact on participants is critical for refining and improving the process [34,35,54,55]. As such, describing the perspectives of all those involved in the development of the CCCE intervention—caregivers, care providers, educators, researchers, and policy leaders—will provide valuable insights into the experiences and recommendations of those participating in co-design processes. Specifically, exploration of participants’ reactions to co-design and their specific critiques and suggestions of it will enable the optimization of future efforts to draw communities together in the service of innovating educational material, practices, policies, and mindsets.

## 2. Materials and Methods

We conducted this cross-sectional study in May–June 2024 after completing the co-design process for the CCCE, descriptions of which have been published elsewhere [45,46]. Our co-design framework integrates process-driven rigor and relational principles, combining methodologies, such as appreciative inquiry, the Hasso Plattner model, and caregiver-centered competencies. A detailed description of the integrated co-design framework is provided in Appendix A.

### 2.1. The Co-Design Team

The full CCCE co-design group included 155 collaborators convened and facilitated by a research team of five. Participation in the co-design team has grown organically over time, driven by trust, momentum, and a shared commitment to person-centered care for caregivers—caregiver-centered care. The process began with a core group of diverse, motivated, multi-level, and interdisciplinary members who initially attended caregiver symposia from 2014 to 2019. This foundational group included caregivers and caregiver champions from various disciplines, such as healthcare, social care, academia, and community organizations.

Following this, the research team expanded the co-design team through iterative recruitment, using a mix of convenience sampling (to understand gaps on the team) and purposeful invitations. Our goal has been to ensure the team reflects unpaid, paid, and professional care and caregiving, representing people of all ages and a variety of caregiving conditions. At all times, at least 25% of the collaborative partners in the co-design group are unpaid caregivers. As the process evolves, we continuously assess representation to identify and address any missing perspectives. To deepen our understanding of caregiving needs and experiences, we conduct additional research exploring the unique challenges faced by double-duty caregivers (balancing paid work and caregiving responsibilities), rural caregivers, and Indigenous caregivers. This ensures that the solutions we co-design address diverse and intersectional caregiving realities, fostering inclusivity and equity.

We prioritize diversity across the following:

- Levels of influence (frontline practitioners, leadership, and policymakers);
- Professional disciplines (healthcare, social care, education, and community sectors);
- Lived experiences (caregivers from diverse communities and caregiving contexts).

To maintain inclusivity and engagement, we periodically review team membership to ensure we have a balanced mix of roles, expertise, and lived experience. This ongoing, adaptive approach allows the co-design team to remain representative, dynamic, and responsive to the evolving needs of the project. It currently includes caregivers (n = 32), team members (n = 5), educational designers (n = 4), healthcare providers (n = 46), health leaders (n = 14), not-for-profit leaders and providers (n = 17), university professors (n = 19), college educators (n = 7), policy makers/ influencers (n = 5) businesspersons (n = 6). Of those, 85 (54.8%) participated in two one-hour meetings on the 6th and 7th of June.

Participants agree to the terms of reference (updated yearly) and provide implied consent by attending meetings. After an introduction to the purpose of the meeting, the 85 participants were deployed into ten focus groups to draw out experiences of the co-design process and elicit suggestions to optimize the co-design process going forward.

### 2.2. Data Collection

Qualitative data, in the form of verbatim transcripts, were collected during ten focus group interviews conducted via Zoom in breakout rooms, each involving six to ten participants. The interviews followed a semi-structured format and were guided by an interview guide designed to explore participants’ experiences with the co-design process, challenges encountered, and recommendations for future involvement. The facilitators used structured prompts (Appendix B) to ensure that discussions captured diverse perspectives and elicited actionable recommendations from participants.

Facilitators, drawing on their expertise in communication, group management, and fostering inclusive co-design spaces, encouraged participants to think critically, collaborate innovatively, and explore new learning approaches. To support thoughtful engagement, the interview guide was shared with participants in advance.

The focus groups lasted 45 to 50 min each. They were digitally recorded and transcribed for later analysis. Identifying details have been omitted in the quotations that are presented below.

### 2.3. Data Analysis

The research team conducted the analysis using Thorne’s interpretive description approach [56,57] and Braun and Clarke’s reflexive thematic analysis [58,59]. We selected this methodology and method for their suitability in health and human-centered research. They are designed to bridge theoretical understanding with practical application. Interpretive description facilitated the exploration of complex, real-world phenomena—such as the co-design process—by generating actionable and contextually relevant insights. This approach allowed the team to identify patterns and themes that illuminated participants’ experiences while grounding findings in the practical realities of caregiving, health and community care practices, and healthcare education [56,57].

Three members of the research team applied Braun and Clarke’s reflexive thematic analysis [50,51] to provide a systematic yet flexible framework for analyzing qualitative data. This method emphasizes reflexivity and iterative coding, enabling the identification of both explicit and underlying meanings within the data. By aligning with the collaborative and evolving nature of the co-design process, this analysis captured the diverse perspectives of the collaborative partners participating in the co-design group. The detailed step-by-step process of the reflexive thematic analysis is outlined in Appendix C. Together, these methodologies generated rich, nuanced themes that informed the development of inclusive and impactful caregiver-centered education.

The research coordinator imported transcripts into NVIVO for ease of group data analysis. The team analyzed the data using Braun and Clarke’s reflexive thematic analysis. The University of Alberta Human Research Ethics Board approved the study protocol Pro00109366 (30 March 2021).

## 3. Results

Participants described the co-design process as transformative and deeply impactful, both personally and professionally. They highlighted significant benefits, identified challenges, and offered recommendations for improving future co-design initiatives. Based on their insights, the findings are grouped into three themes: growth through collaboration and inclusivity, challenges and opportunities for improvement, and recommendations for enhancing future co-design processes.

### 3.1. Growth Through Collaboration and Inclusivity

The co-design process fostered a unique environment of collaborative learning and inclusivity, enabling participants to expand their understanding, build skills, and connect with diverse perspectives. The group’s diversity—spanning caregivers, healthcare professionals, educators, and policymakers—broadened participants’ viewpoints and inspired innovative solutions. Participants reported that exposure to varied perspectives challenged their usual ways of thinking, encouraging reflection on their professional practices. One participant noted, *“Every time I join these meetings, it makes me reflect on, ‘How do I consider caregivers in all the processes that I do?’—in the policy process and in programming”*.

The discussions also deepened participants’ appreciation of the lived experiences of caregivers. As another participant shared, “I always went away learning something. That’s what really made the process engaging and the product exceptional”.

Inclusivity was a cornerstone of the co-design process, fostering a sense of equity and validation. Participants frequently praised the collaborative environment, emphasizing that all voices—regardless of background, expertise, or role—were heard and valued. One participant explained, *“This has been one of the most inclusive processes I’ve been involved with. The validation and value of every participant have been so clear”*.

Participants also credited the success of the co-design process to skilled leadership and facilitation. Leaders ensured that discussions remained respectful and productive while fostering collaboration. A participant highlighted, *“The facilitators ensured everyone’s voice was heard, creating a collaborative and productive environment”*. Our facilitators, with diverse professional backgrounds—including a physician, nurse, gerontologist, dietician, and educational designers—greatly enrich the co-design process by bringing unique perspectives, skills, and expertise. We believe that our multidisciplinary team fosters a comprehensive and innovative approach, enabling solutions that consider multiple facets of the caregiving experience. Their differing priorities and expertise help illuminate blind spots and address gaps that might otherwise be overlooked.

As facilitators, our goal is to minimize bias and ensure that everyone’s voices are heard in the breakout rooms. Additionally, facilitators review recordings and reflect on how they engaged participants, the percentage of time they spent talking compared to participants, and how their own assumptions, language, and behaviors may have influenced the discussions.

By integrating diverse perspectives and maintaining an inclusive atmosphere, the co-design process empowered participants to advocate more effectively for caregivers within their own professional spheres. Quantitatively, more than 70% of participants reported increased confidence in considering caregiver needs in their work, and nearly two-thirds felt motivated to initiate caregiver-centered changes in their organizations.

### 3.2. Challenges and Opportunities for Improvement

Despite its overall success, participants identified challenges in the co-design process related to information management, time constraints, and the size of the group.

- Information Overload: Many participants found it difficult to process the volume of information shared at the start of meetings, describing it as overwhelming. One participant remarked, *“There’s such a brain dump at the beginning. I couldn’t follow along… I was looking through my emails to see if there was something attached, I could use to keep up”*.
- Time Management: Balancing participation in the co-design group with personal and professional commitments was another challenge. Many reported it was challenging to find time in busy workdays, especially in the current shortage of health providers. One participant noted, *“I couldn’t attend all the meetings because I was busy… It was daunting at times to keep up”*.
- Large Group Size: While the group’s size brought valuable diversity, it also made discussions more complex. Participants appreciated strategies like breakout rooms, which allowed for smaller, focused discussions. A participant shared, *“Having such a large group gave us a wide perspective, but it was also cumbersome. The breakout rooms helped manage this well”*.

These challenges highlighted areas for process improvement, emphasizing the need for better information sharing, flexible scheduling, and smaller group interactions.

### 3.3. Recommendations for Enhancing Future Co-Design Processes

Participants provided the following actionable suggestions to optimize future co-design initiatives:

- Pre-Meeting Preparation: Participants suggested providing concise, plain-language summaries and pre-meeting materials to reduce information overload. One participant remarked, *“If we could get some point form or easy-to-read overviews along with all the information at the beginning—it would help us follow discussions better”*.
- Flexible Scheduling: To accommodate participants’ diverse workloads, they recommended offering meetings on different days and spacing them at least a week apart.
- Leveraging Technology: Participants emphasized the importance of improving accessibility for caregivers in remote or underserved areas. As one participant explained, *“How do we bring this education to remote areas with limited internet access? This needs to be addressed if we want it to reach everyone”*.
- Diverse Stakeholder Inclusion: Participants stressed the importance of including a broad range of voices, including rural caregivers, immigrant communities, and frontline healthcare providers. One participant shared, *“The strongest thing is having caregivers in the mix to keep us grounded on what we’re focused on—what they really need and want”*.
- Participants also emphasized the importance of engaging policymakers to align strategies with broader healthcare systems and funding mechanisms. Another participant noted, *“You need frontline healthcare providers in the design process to ensure the strategy reflects real-world needs”*.

By addressing these recommendations, future co-design initiatives can better support diverse participants, enhance engagement, and foster more impactful outcomes.

The co-design process provided participants with transformative opportunities for learning, collaboration, and advocacy. By fostering an inclusive environment with strong facilitation and diverse perspectives, the process enabled participants to co-create meaningful and actionable solutions for caregiver-centered care. Despite challenges with information management, time constraints, and group size, participants’ recommendations provide a roadmap for optimizing future co-design initiatives. With adjustments to preparation, scheduling, and stakeholder inclusion, co-design can continue to drive systemic change in caregiver support and education.

## 4. Discussion

This study explored the experiences of participants engaging in a co-design process to develop caregiver-centered education for healthcare providers. The co-design process offered participants transformative opportunities for learning, collaboration, and advocacy, supported by a structured yet flexible framework (Appendix A). By cultivating an inclusive environment with skilled facilitation and diverse perspectives, the process empowered participants to co-create meaningful and actionable solutions for caregiver-centered care. While challenges such as information management, time constraints, and group dynamics emerged, participants provided valuable recommendations to optimize future initiatives. Enhanced preparation, flexible scheduling, and broadened stakeholder inclusion were identified as key strategies to strengthen the process, ensuring co-design remains a powerful driver of systemic change in caregiver support and education.

### 4.1. Contributions to the Literature on Co-Design

The findings of this study are consistent with the existing literature on the benefits of co-design, particularly its ability to validate participant contributions, enhance engagement, and ensure the relevance of solutions to practical contexts [34,60]. The application of co-design within this initiative, guided by the Kirkpatrick Barr education framework [46], resulted in measurable increases in participants’ knowledge, skills, and confidence to engage effectively with caregivers. These findings echo conclusions from systematic reviews [34,60] that highlight co-design’s ability to bridge the gap between lived experiences and professional practice.

This study extends current understanding by emphasizing how co-design promotes inclusivity and mitigates systemic inequities in caregiver-centered healthcare education. While prior studies [34,35] have identified the value of co-design in fostering trust and innovation, this research highlights specific strategies—such as flexible scheduling and streamlined information delivery—that can enhance the accessibility and effectiveness of co-design initiatives. Furthermore, the integration of culturally diverse and marginalized voices in this process underscores the importance of targeted outreach and recruitment in achieving equitable outcomes.

### 4.2. Practical Implications for Co-Design Processes

The intentional recruitment of a diverse participant group, including caregivers, healthcare providers, educators, and policymakers, was central to this initiative. Nearly one-third of participants were current or former caregivers, ensuring that lived experiences shaped the development of education. Despite these efforts, representation gaps among Indigenous, immigrant, and rural caregivers persisted, emphasizing the need for more inclusive recruitment strategies. This finding aligns with Peters [34] and Karlsson [61], who stress that achieving diversity in co-design enriches outcomes while addressing barriers to participation.

Our research with underserved caregiver populations [15,16,61] and flexible participation to accommodate intensive caregiving or work schedules has helped us to develop relationships and increase our co-design team’s diversity. Additional efforts, such as partnering with community organizations that serve Indigenous, immigrant, and rural populations, and offering culturally appropriate engagement strategies, could further enhance representation and inclusion on the co-design team.

Logistical challenges emerged as another area requiring refinement. Reported difficulties with information overload at the start of meetings, which hindered their ability to engage fully in discussions. Simplifying pre-session materials into point-form summaries was recommended to reduce cognitive load and enhance understanding. This recommendation is supported by Avila-Garzon [35], who notes that effective information delivery is critical to sustaining participant engagement. Summaries and plain language material should also increase accessibility for equity deserving participants.

Time constraints also presented significant barriers, with participants balancing work, family, and caregiving responsibilities. Flexible scheduling, such as alternating meeting days and spacing sessions to avoid overlap with holidays or work shifts, was identified as an effective solution. Similarly, the use of breakout rooms to manage large group dynamics proved valuable in fostering deeper engagement and generating richer insights. These strategies reflect best practices in co-design facilitation, as noted by Karlsson [60], who emphasizes the importance of small, focused discussions in optimizing participant contributions.

### 4.3. Leadership and Facilitation in Co-Design

Effective leadership emerged as a pivotal factor in the success of the co-design process. Facilitators played a critical role in creating an inclusive environment that addressed power imbalances and ensured all participants felt valued and respected. This aligns with findings from Peters [34] and Avila-Garzon [35], who emphasize the importance of skilled leadership in fostering collaboration and maintaining engagement. Participants highlighted the facilitators’ ability to validate contributions, sustain momentum, and encourage quieter voices, demonstrating the importance of thoughtful leadership in achieving meaningful outcomes.

### 4.4. Barriers and Opportunities for Improvement

While the co-design process successfully engaged a wide range of stakeholders, challenges related to representation and logistics highlighted areas for improvement. The limited inclusion of underrepresented groups, such as Indigenous and rural caregivers, underscores the need for culturally sensitive recruitment strategies. Additionally, managing large group dynamics and addressing information overload are critical to ensuring that participants can contribute effectively. These findings suggest that co-design teams should prioritize strategies that enhance accessibility, such as simplifying preparatory materials, leveraging hybrid meeting formats, and scheduling sessions to accommodate diverse participant needs.

### 4.5. Advancing Co-Design: A Model for Inclusive Education

This study demonstrates how co-design fosters collaboration by integrating diverse perspectives and lived experiences into actionable solutions. By addressing logistical and structural barriers, co-design teams can create processes that are both inclusive and impactful. Importantly, the findings emphasize the value of diversity in enriching discussions, preventing tunnel vision, and ensuring that solutions reflect the varied realities of caregiving. These insights reinforce the transformative potential of co-design as a tool for driving systemic change in healthcare education.

Researchers often find knowledge translations onerous [62]. Co-design facilitates integrated knowledge translation, bridging the gap between research and practice. For practitioners, this ensures that education and interventions are relevant, actionable, and inclusive [31]. For policymakers, co-design offers a framework to develop equitable, community-centered policies that address systemic barriers and amplify diverse voices.

### 4.6. Limitations and Future Directions

One limitation of this study was the lack of established co-design frameworks at its inception, which required the team to adapt and innovate in real time. This iterative approach, while necessary, may have resulted in inefficiencies during early phases. Additionally, despite efforts to recruit a diverse participant pool, representation gaps highlight the ongoing need for targeted outreach to include underserved groups, particularly Indigenous and immigrant caregivers.

Future research should focus on addressing these challenges and exploring the long-term impacts of co-design on participants and healthcare systems. Longitudinal studies could examine whether co-design initiatives sustain engagement, influence organizational culture, or drive systemic improvements in caregiver support. Furthermore, integrating robust feedback mechanisms into co-design processes would enable teams to evaluate and refine their approaches continuously, ensuring ongoing relevance and effectiveness.

## 5. Conclusions

This study demonstrates the transformative impact of the co-design process on participants and the development of CCCE. The findings emphasize the value of inclusivity and collaboration in co-design. By involving diverse collaborative partners—including caregivers, not-for-profit associations such as caregiver and disease-specific or condition-focused organizations, healthcare providers, educators, and policymakers—the process ensures that solutions are reflective of lived experiences and tailored to meet the real-world needs of caregivers and care recipients.

This study calls on practitioners, educators, and policymakers to adopt co-design approaches and prioritize caregiver-centered initiatives. By investing in participatory methods and supporting further research, collaborative partners can help transform healthcare systems to be more responsive and inclusive. Such efforts are crucial for ensuring that caregivers are recognized and supported, ultimately improving the well-being of caregivers, care recipients, and the broader healthcare ecosystem.

## Figures and Tables

**Figure A1:**
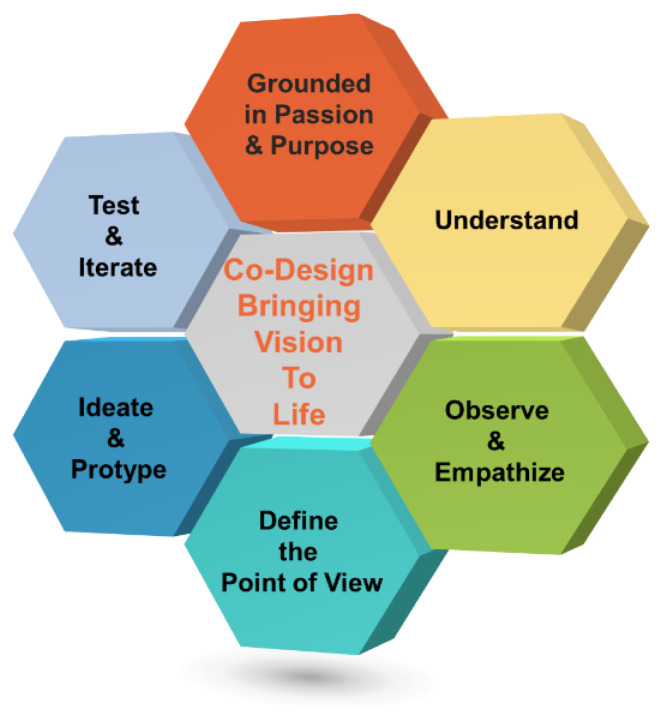
Caregiver-Centered Care Co-design Framework: Bringing Vision to Life.

## Data Availability

Data are available from the authors upon request.

## Abbreviations

The following abbreviations are used in this manuscript:

CCCE	Caregiver-centered care education

## Appendix A. Integrated Co-Design Framework: From Understanding to Real-World Solutions

### Appendix A.1. Bringing Caregiver-Centered Solutions to Life

This framework represents an evolving process designed to make caregivers and their roles visible and valued. Expert facilitation and continuous feedback from our 155-person multidisciplinary co-design team have driven systemic and cultural change. Drawing from Bill Bannear’s concept of the four voices of design thinking [63], the team integrates:

- Voices of Intent: Members with passion, motivation, or authority to catalyze change.
- Voices of Capability: Those contributing resources, expertise, or access to critical spaces, such as worksites.
- Voices of Experience: Family caregivers, healthcare and social care providers, and stakeholders who live caregiving or are affected by the outcomes.
- Voices of Design: Educational designers from The Learning Design Studio at the University of Alberta, who brokered and facilitated module creation and coordination.

This framework synthesizes diverse expertise, lived experiences, and resources to co-create meaningful, impactful solutions for caregivers.

### Appendix A.2. Caregiver-Centered Co-Design Framework: Vision to Life for Relational Care and Systems Transformation

Our co-design framework is iterative and integrates process-driven rigor (understand, observe, dawn, ideate, prototype, test) of the Hasso Plattner model [64,65,66], relational principles of appreciative inquiry [67,68], and person-centered care [69,70]. The steps align with caregiver-centered competencies [44], such as recognizing strengths, communicating with empathy, fostering solutions, and enhancing outcomes. This blend of methodologies ensures the conditions for meaningful solutions to emerge, transforming care systems and cultures.

1. Grounded in Passion and Purpose

**Objective**: Build shared commitment to transforming the culture and context of care.
**Key Elements**: Authentic relationships, inclusion, interdisciplinary synergy, and shared purpose.
**Activities**: Cultivate collaboration through humble leadership, shared vision, and the Caregiver-Centered Care Competency Framework as the “North Star”.

2. Understand

**Objective**: Grasp the “care” space and build contextual understanding.
**Activities**: Conduct literature reviews, synthesize insights, and plan research.
**Relevance**: Establishes a foundation for relationship-based, person-centered design.

3. Observe and Empathize

**Objective**: Gather diverse perspectives and lived experiences.
**Activities**: Use interviews, case studies, immersion, and secondary data analysis.
**Relevance**: Promotes empathy and inclusion.

4. Define the point of view

**Objective**: Synthesize insights into a clear, actionable perspective.
**Activities**: Analyze findings, prioritize user needs, and craft focus areas.
**Relevance**: Aligns insights with user-centered goals and maps the path forward.

5. Ideate and Prototype

**Objective**: Generate and test creative solutions.
**Activities**: Brainstorm ideas, categorize by feasibility, and develop prototypes.
**Relevance**: Encourages collaboration, rapid iteration, and actionable outcomes.

6. Test and Iterate

**Objective**: Refine solutions through user feedback and continuous learning.
**Activities**: Conduct usability testing, integrate feedback, and make iterative improvements.
**Relevance**: Ensures solutions are validated and impactful in real-world contexts.

### Appendix A.3. Building the Future of Care

By integrating process rigor with relational and participatory principles, this co-design framework creates a balance between structured action and creative exploration. It fosters inclusive, person-centered solutions that address systemic challenges, ultimately driving cultural and systemic transformation in caregiving.

## Appendix B. Focus Group Interview Guide

1. What did you think about the experience of participating in the co-design process?

What were the best parts?
What was least enjoyable?

2. What challenges do you think there were in this co-design process?

What would you like to have changed?

3. Who needs to be involved in the co-design group?

Who is missing?

## Appendix C. Steps in Reflexive Thematic Analysis

We used the Consolidated Criteria for Reporting Qualitative Research (COREQ) in this report [71],

1. Familiarization with the Data:
  - Listen to the Interviews.
  - Correct and verify the transcriptions when listening to the interviews.
  - Read and re-read the data to immerse yourself fully.
  - Actively engage with the data to understand the depth and breadth of the content.
  - Note initial impressions, ideas, or patterns as you review the material.
2. Generating Initial Codes:
  - Systematically identify and label features of the data that are relevant to the research question.
  - Assign codes to meaningful segments of the data (e.g., words, phrases, sentences).
  - Codes can be semantic (explicit meanings) or latent (underlying ideas or assumptions).
  - Use an iterative and flexible process, adding or refining codes as necessary.
3. Searching for Themes:
  - Group similar or related codes together to identify broader patterns or themes.
  - Themes are conceptual groupings that capture something significant about the data.
  - Focus on relationships between codes, organizing them into initial themes and sub-themes.
  - This step involves moving beyond the data to interpret its meaning.
4. Reviewing Themes:
  - Refine and adjust themes by reviewing them against the coded data and the entire dataset.
  - Ensure themes are coherent, distinct, and supported by the data.
  - Some themes may merge, split, or be discarded at this stage.
  - Develop a thematic “map” to visualize the relationships between themes and sub-themes.
5. Defining and Naming Themes:
  - Articulate the essence of each theme by identifying what it captures and why it is significant.
  - Provide detailed descriptions that explain the story each theme tells in relation to the research question.
  - Name themes in a way that succinctly conveys their core meaning.
6. Writing the Report:
  - Present the themes and their supporting data in a way that answers the research question and tells a coherent story.
  - Use data extracts (e.g., quotes) to illustrate themes, ensuring they are contextualized and meaningful.
  - Provide a clear narrative that integrates your analytic interpretations with the data.
